# Case Report: Maximizing the anti-proteinuric response: a multicenter real-world sparsentan case series in IgA disorders

**DOI:** 10.3389/fneph.2025.1673799

**Published:** 2025-11-21

**Authors:** Abhisekh Sinha Ray, Praveen Errabelli, Maulik Lathiya, Neeharik Mareedu

**Affiliations:** 1 Division of Nephrology and Critical Care, Creighton University, Omaha, NE, United States; 2 Division of Nephrology, Allina Health, Minneapolis, MN, United States; 3 Division of Nephrology, Mayo Clinic, Rochester, MN, United States; 4 Division of Nephrology, UPMC Western Maryland, Cumberland, MD, United States

**Keywords:** IgA nephropathy, sparsentan, IgA vasculitis, proteinuria, glomerulonephritis

## Abstract

Immunoglobulin A Nephropathy (IgAN) is a prevalent form of glomerulonephritis that leads to chronic kidney disease (CKD), typically marked by ongoing proteinuria, even when treated with standard therapies such as renin-angiotensin-aldosterone system (RAAS) blockers and occasionally immunosuppression. Proteinuria is a modifiable risk factor crucial for disease advancement. Sparsentan, a dual endothelin receptor and angiotensin receptor blocker (DEARA), has been introduced as a novel therapeutic option focusing on proteinuria. We present a case series featuring seven patients - five diagnosed with IgAN and two with IgA vasculitis (IgAV) - with severe proteinuria who were treated with Sparsentan, sometimes in combination with other medications such as targeted-release formulation (TRF) budesonide, sodium-glucose cotransporter-2 (SGLT2) inhibitors, or mycophenolate. Notable reductions in proteinuria and improvements in blood pressure regulation were observed in these cases. Sparsentan was well-tolerated overall, with no significant hyperkalemia or hepatotoxicity reported in this group. These cases emphasize the real-world experience, promising efficacy and safety of Sparsentan in reducing proteinuria in patients with IgA-mediated glomerular disorders, including its application in combination therapies and patients with concurrent or prior immunosuppression

## Introduction

IgAN is the most common primary glomerulonephritis globally and a major contributor to kidney failure (1). The disease’s development follows a multi-hit model, usually starting with the generation of galactose-deficient Immunoglobulin A1, which leads to the formation and deposition of immune complexes in the glomerular mesangium, triggering inflammation and damage (2). IgAV, formerly known as Henoch-Schönlein purpura, may also affect the kidneys and exhibits similar histological features, presenting a risk for progressive renal disease (3). Chronic proteinuria significantly increases the likelihood of advancing to end-stage kidney disease in these individuals (4), and risk stratification often incorporates clinical factors as well as histological characteristics from the Oxford classification (MEST-C score) (5).

Standard management focuses on comprehensive supportive care and targeted therapies primarily designed to lower proteinuria and control blood pressure (6). Supportive strategies encompass lifestyle changes such as limiting dietary sodium to less than 2 grams per day, maintaining a healthy weight, quitting smoking, and avoiding nephrotoxins. Maintaining strict blood pressure control is essential, with typical targets set below 130/80 mmHg, and potentially lower—at 125/75 mmHg—in patients with significant proteinuria greater than 1 gram per day, if feasible (6). The foundational approach to pharmacologic treatment aimed at reducing proteinuria involves blocking the RAAS with the highest tolerable doses of either angiotensin-converting enzyme inhibitors (ACEi) or angiotensin II receptor blockers (ARBs) (6). For those at high risk of disease progression, such as those showing persistent proteinuria of 1 gram per day or more (acknowledging that the optimal proteinuria threshold for treatment escalation remains a subject of ongoing debate, with goals ranging from <1.0 g/day to <0.5 g/day in different guidelines) despite 3 to 6 months of optimized supportive care and RAAS blockade or in the patients with active proliferative lesions or crescents or the patients with evidence of worsening kidney function, systemic corticosteroids or other immunosuppressants may be contemplated (6, 7). Nonetheless, any potential benefits must be carefully weighed against substantial risks, as evidenced in trials like TESTING (6, 7). Despite these interventions, many patients still experience ongoing proteinuria, underscoring the necessity for further therapeutic interventions (7).

Activation of endothelin-1 (ET-1), associated with podocyte injury, mesangial cell proliferation, inflammation, and fibrosis (8, 9), is another potential therapeutic target. Sparsentan is the first dual antagonist of the endothelin type A receptor (ETAR) and the angiotensin II type 1 receptor (AT1R) (9). By inhibiting both pathways, Sparsentan may provide a more effective antiproteinuric effect compared to RAS blockade alone (9). The Phase 3 PROTECT trial showed that Sparsentan was more effective than irbesartan in lowering proteinuria in patients with IgAN (10). Furthermore, the use of Sparsentan in combination with other agents, such as targeted-release budesonide for improved proteinuria reduction in advanced IgAN, is an area of ongoing investigation (11, 12). Here, we present seven cases illustrating the use of Sparsentan therapy and its outcomes in patients with IgAN and IgAV nephritis treated in clinical practice.

## Case presentations

### Case 1

A 40-year-old morbidly obese male presented with proteinuria (1.7 g/day), hematuria, and renal insufficiency (Creatinine [Cr] 1.3 mg/dL). A renal biopsy confirmed IgAN (Oxford Classification: M1,E0,S1,T0,C0) with 15% interstitial fibrosis and tubular atrophy (IFTA). Initial steroid therapy (Pozzi protocol) reduced the urine protein/creatinine ratio (UPCR) to 0.77 g/g. He was maintained on maximal tolerable dose of Losartan. Four years after the initial steroid treatment, he experienced another acute flare (UPCR 1.6 g/g; Cr 1.5 mg/dL), which partially responded to a second course of steroids (UPCR 0.7 g/g). A repeat kidney biopsy was not pursued due to the patient’s reluctance for further invasive procedures, and the decision was made to intensify pharmacologic therapy based on clinical and laboratory evidence of persistent active disease. As he declined further immunosuppression, he was treated with a maximal tolerable dose of Losartan, Finerenone (nonsteroidal mineralocorticoid receptor antagonist [MRA]), and Empagliflozin (SGLT2 inhibitor). Despite these measures, blood pressure (BP) remained suboptimally controlled, and UPCR was 0.57 g/g. Losartan and Finerenone were discontinued, and the patient was started on Sparsentan. After 7 months of treatment with Sparsentan, Cr remained stable at 1.4 mg/dL, UPCR improved markedly to 0.1 g/g and BP was well-controlled. No Sparsentan-related adverse events were noted.

### Case 2

A 64-year-old male with a history of hypertension, atrial fibrillation, coronary artery disease, and chronic microscopic hematuria was referred after a skin biopsy confirmed leukocytoclastic vasculitis. He presented with edema, a recurring rash that initially responded to steroids, and abdominal pain. Urinalysis indicated 2+ blood and 1+ protein (UPCR 0.6 g/g; creatinine was 1.1 mg/dL), and blood pressure remained suboptimally controlled on maximal tolerable dose of Losartan. A renal biopsy demonstrated IgA-dominant mesangial deposits and 15% IFTA, consistent with IgAV nephritis (Oxford classification could not be determined). Losartan was discontinued, Sparsentan was started, prednisone (for vasculitis) was tapered over 6 weeks at the time of Sparsentan initiation as the cutaneous vasculitis symptoms were resolving, and SGLT2 inhibitor was added. After 9 months on Sparsentan, urinalysis became bland, UPCR improved to 0.05 g/g, creatinine normalized to 0.87 mg/dL, and blood pressure was well controlled. No adverse events related to Sparsentan were observed.

### Case 3

A 63-year-old woman with hypertension, hyperlipidemia, and recurrent UTIs presented with sub-nephrotic proteinuria (UPCR 1.9 g/g increasing to 2.1 g/g despite being on maximal tolerable dose of Losartan) and hematuria (3+ blood, dysmorphic RBCs), while her creatinine levels remained normal. Her blood pressure was appropriately controlled. Renal biopsy confirmed IgAN (Oxford classification: M1,E0,S1,T0,C0) with 10-15% global glomerulosclerosis and 10-15% IFTA). Losartan was discontinued, and treatment with Sparsentan 200 mg daily was started, in addition to SGLT2 inhibitor. After 8 months, her renal function remained normal, urine sediment showed no abnormalities, and proteinuria resolved (spot urine protein <6 mg/dL, UPCR not calculable). Blood pressure continued to be well-controlled, and no adverse events related to Sparsentan were observed.

### Case 4

A 36-year-old male with hypertension and nephrolithiasis presented with worsening renal function (baseline Cr 1.6−2.0 mg/dL), proteinuria (UPCR 2.0 g/g), hematuria, and poorly controlled hypertension. Renal biopsy showed IgAN (Oxford classification: M1,E0,S1,T1,C0) with significant chronicity (50% global glomerulosclerosis, 50% IFTA). Losartan was discontinued, Sparsentan was started (titrated to 400 mg daily), and SGLT2 inhibitor was added. After 6 months, UPCR improved to 0.6 g/g although Cr fluctuated (2.0−2.3 mg/dL). No adverse events related to Sparsentan were observed.

### Case 5

A 47-year-old male with type 2 diabetes, chronic hypertension, morbid obesity, and a history of tobacco use presented with proteinuria, hematuria, and a maculopapular rash. Renal biopsy revealed IgAN (Oxford Classification: M1,E1,S0,T0,C1) with mild mesangial cellularity, endocapillary hypercellularity, cellular crescents, and 15% IFTA. Baseline serum creatinine was 0.8 mg/dL. Initial UPCR was 4 g/g with moderate hematuria (13 RBCs/hpf). While GLP-1 inhibitors are valuable for weight management and cardiovascular risk reduction, the primary and most urgent clinical driver for this patient was the rapid progression of IgAN and nephrotic-range proteinuria (4 g/g) with active crescents (C1 lesion). Therefore, he was started on concurrent therapy with TRF budesonide and Sparsentan, targeting both the inflammation and the hemodynamic/podocyte injury pathways simultaneously. In the first 6 weeks, proteinuria worsened to 7.8 g/g and hematuria increased (32 RBCs/hpf). After 9 months of combination therapy, UPCR improved to 0.1−0.2 g/g. At this point, Tarpeyo was discontinued, and the patient continued on Sparsentan with an SGLT2 inhibitor. Over the next 6 months on therapy, UPCR decreased from around 0.17 g/g to 0.07 g/g. No adverse events were noted during treatment.

### Case 6

A 34-year-old male with chronic hypertension, class II obesity, and obstructive sleep apnea presented for ongoing proteinuria associated with his history of diffuse proliferative necrotizing IgA glomerulonephritis in the setting of IgAV (Henoch-Schönlein purpura), diagnosed around age 15. Initially, he underwent steroid treatment at diagnosis and was subsequently maintained on the highest tolerable dose of Irbesartan and mycophenolate for maintenance immunosuppression. The use of mycophenolate in this patient reflects a historical or region-specific choice for a steroid-sparing agent in a patient with a severe, necrotizing form of IgAV nephritis diagnosed at a young age, even while acknowledging its controversial role in non-Chinese IgAN populations. His serum Cr levels were stable between 0.8−0.9 mg/dL. However, proteinuria worsened from 0.6 g/g to 2.5 g/g despite continued treatment with Irbesartan and mycophenolate (Oxford score unavailable). Given the clear clinical and laboratory evidence of worsening disease (2.5 g/g proteinuria) and the availability of a new, non-immunosuppressive therapeutic agent (Sparsentan), the clinical decision was made to escalate therapy based on the established diagnosis of IgAV nephritis and the need for greater proteinuria reduction, without requiring a repeated biopsy after 19 years. Irbesartan was stopped, and he was started on Sparsentan. After 8 months on Sparsentan (in combination with mycophenolate), his UPCR markedly decreased to 0.78 g/g, while serum Cr level remained stable (0.8−0.9 mg/dL). No side effects related to Sparsentan were observed.

### Case 7

A 30-year-old woman with a history of chronic hypertension and migraines presented with uncontrolled blood pressure. Diagnostic tests indicated elevated serum creatinine (1.4−1.7 mg/dL), nephrotic-range proteinuria (UPCR 5.1 g/g), and hematuria (large blood, with 15 RBCs/hpf). A renal biopsy confirmed the diagnosis of IgAN (Oxford classification: M0,E0,S1,T0,C1), showing 13 out of 32 glomeruli globally sclerotic, cellular crescents, mild expansion of the mesangial matrix, no endocapillary hypercellularity or fibrinoid necrosis, and 10% IFTA. The patient was initiated on a treatment regimen including Sparsentan and SGLT2 inhibitor. After four months of this therapy, her UPCR showed substantial improvement, decreasing from 5.1 g/g to 0.75 g/g. Serum Cr levels remained stable (1.4−1.7 mg/dL) and there were no noted side effects related to Sparsentan.

## Discussion

This case series illustrates the potential effectiveness of Sparsentan in reducing proteinuria in patients with IgA-mediated kidney disease, including IgAN and IgAV, in a real-world clinical context. All seven patients experienced a decrease in proteinuria after starting Sparsentan, often used in combination with other treatments, as detailed in **Table 1**. Importantly, substantial antiproteinuric effects were noted in those with ongoing proteinuria, despite previous treatments with ARBs (Patients 1-4, 6), SGLT2 inhibitors (added concurrently or previously in patients 1, 3, 4, 5, 7), mineralocorticoid receptor antagonists (Patient 1), or corticosteroids/immunosuppressants (Patients 1, 2, 4, 5, 6). A central theme across all cases is the persistence of proteinuria despite maximized supportive therapy (RAAS blockade, optimized blood pressure, and in patient 1, a nonsteroidal MRA). Our clinicians’ decision to introduce Sparsentan was driven by this residual proteinuria, demonstrating a commitment to intensifying treatment *after* standard supportive care had been optimized, aligning with current guidelines and the necessity for new therapeutic classes. The simultaneous use of SGLT2 inhibitors in five patients is noteworthy, as these medications have shown considerable advantages in reducing CKD progression across various etiologies, including IgAN (13).

**Table 1:** Table representing patients proteinuria response and treatments they received.

Patient Number, Age/Sex/Diagnosis	Oxford MEST-C Score	Baseline P/C ratio (g/g)^1^	Post Sparsentan P/C ratio (g/g)^2^	Duration of Sparsentan Therapy (months)	Absolute reduction of P/C ratio	% reduction in proteinuria^4^	Steroid use relative to Sparsentan therapy	Concomitant SGLT2i use	Further comments
Patient 1: 40/M (IgAN)	M1E0S1T0C0	0.57	0.1	7	0.47	82.5%	Prior steroids completed	Yes	Received prior steroid courses (last one ended months before Sparsentan initiation)
Patient 2 64/M (IgAV/IgAN)	N/A	0.6	0.05	9	0.55	91.7%	Prednisone tapered off shortly after start	Yes	Prednisone taper (for IgAV) over 6 weeks shortly after Sparsentan initiation
Patient 3: 63/F (IgAN)	M1E0S1T0C0	2.1	<0.06^3^	8	>2.04	>97%	None	Yes	No steroid therapy reported. Post-Sparsentan proteinuria <6 mg/dL (P/C <0.06)
Patient 4: 36/M (IgAN)	M1E0S1T1C0	2.0	0.6^4^	6	1.4	70%	None	Yes	6 months on Sparsentan + SGLT inhibitor
Patient 5: 47/M (IgAN)	M1E1S0T0C1	4.0	0.07*^5^*	15	3.93	98.3%	Budesonide added *concurrently*	No	Initial worsening (UPCR 7.8 g/g). On Sparsentan & Tarpeyo for 9 months (UPCR 0.1 g/g), then Tarpeyo stopped. Final UPCR 0.07 g/g after 6 months
Patient 6: 34/M (IgAV/IgAN)	N/A	2.5	0.78	8	1.72	68.8%	On Mycophenolate as steroid sparing agent	No	H/o HSP/IgAV (necrotizing) since age 15. Worsening UPCR (0.6 g/g to 2.5 g/g) despite mycophenolate. Sparsentan added with reduction in UPCR
Patient 7: 30/F (IgAN)	M0E0S1T0C1	5.1	0.75	4	4.35	85.3%	None reported	Yes	Nephrotic-range proteinuria (UPCR 5.1g/g) with crescents (C1). Started Sparsentan + SGLT2 Inhibitor. Cr stable

^1^ Pre-Sparsentan Urine protein/creatinine ratio: Value immediately before initiating Sparsentan (or concurrent therapy including Sparsentan for Pt 5).

^2^ Post-Sparsentan Urine protein/creatinine ratio: Value achieved after the specified duration on Sparsentan (+/- SGLT2i, +/- steroids/IS as noted).

^3^ Patient 3 Post-Value: Proteinuria reduced below the limit of detection (<6 mg/dL).

^4^ Percentage Reduction: Calculated as ((Pre-Sparsentan P/C) - (Post-Sparsentan P/C)) / (Pre-Sparsentan P/C) * 100%. Calculation for Pt 5 uses final Post-Sparsentan value.

^5^ Patient 5 Post-Value: Final value after 9 months of Sparsentan+Tarpeyo, followed by 6 months of Sparsentan+SGLT2i.

N/A: Not Available/Applicable.

For both Patients 2 and 7, where SGLT2 inhibitors and Sparsentan were initiated concurrently, we acknowledge the potential for confounding, as both agents contribute substantial antiproteinuric and renoprotective effects. However, this combined approach reflects the real-world clinical strategy of rapidly introducing multiple, non-overlapping mechanisms of action in patients at high risk of progression (e.g., IgAV nephritis in patient 2, nephrotic range proteinuria in patient 7). The profound reduction in proteinuria in these patients, a key outcome of both the PROTECT and EMPA-KIDNEY trials, supports the clinical rationale for this synergistic, multi-targeted regimen. These findings align with results from the pivotal Phase 3 PROTECT trial, which compared Sparsentan to an active comparator, irbesartan (an ARB), in patients with IgAN and persistent proteinuria (≥1 g/day) despite optimized RAAS blockade (10). The trial met its primary efficacy endpoint at the interim analysis, demonstrating a statistically significant and clinically meaningful greater reduction in proteinuria from baseline after 36 weeks of treatment with Sparsentan compared to Irbesartan (mean reduction of 49.8% vs. 15.1%, respectively) (10). The two-year analysis confirmed these findings and showed a favorable effect on the secondary endpoint of estimated glomerular filtration rate (eGFR) slope, indicating slower kidney function decline with Sparsentan compared to Irbesartan over the long term. Our case series, while small, reflects this potential for substantial proteinuria reduction, even in patients previously treated with multiple agents and across a range of baseline proteinuria levels, including nephrotic range (Cases 5 and 7).

The inclusion of patient 2 and 6 with IgAV nephritis suggests that Sparsentan’s potential benefits span the spectrum of IgA-mediated glomerular diseases. The growing body of evidence supporting this is underscored by recent reviews that have identified endothelin receptor antagonists, alongside SGLT2 inhibitors and other agents, as promising potential future therapies for IgAV nephritis (14). The heterogeneity of our cohort, reflected in the varied Oxford MEST-C scores (where available) and the presence of crescents in only a minority of cases (Patients 5 and 7), underscores the wide spectrum of IgA disorders encountered in routine clinical practice. Sparsentan’s efficacy was apparent even in patients without active proliferative features, aligning with its proposed anti-fibrotic and hemodynamic benefits which extend beyond just active inflammation. Patient 7, presented with nephrotic-range proteinuria and crescents (C1), achieved a rapid and substantial (>85%) reduction in proteinuria within 4 months of starting Sparsentan with an SGLT2 inhibitor, all while maintaining stable creatinine levels.

The dosing of Sparsentan, primarily 200 mg daily with titration to 400 mg only in patient 4, was a clinician’s choice based on a personalized, risk-benefit assessment. The decision to use the maximal dose is often reserved for patients with more severe or refractory proteinuria, as was the case for patient 4 with his significant chronic changes.

Patient 4 illustrates the difficulties in managing IgAN with significant chronic changes (50% IFTA, T1 lesion). Sparsentan was still effective in reducing proteinuria in this patient by 70%. Case 5 highlights the challenges of starting Sparsentan and budesonide simultaneously, a treatment demonstrated in the Phase 3 NeflgArd trial to decrease proteinuria and slow the decline of eGFR in IgAN (15). Reviews discussing the evolving landscape of IgAN therapy suggest that combination treatment with agents targeting different pathogenic pathways, such as TRF-budesonide and sparsentan, is a logical approach and will likely be central to future management strategies (16). The potential for initial combination therapy with TRF-budesonide and Sparsentan is also being explored, as highlighted in emerging clinical experience and case reports (17, 18). Our case showed an initial unexpected rise in proteinuria before a substantial improvement, ultimately showing lasting benefits with Sparsentan combined with an SGLT2 inhibitor after discontinuing budesonide. These examples emphasize the variability in IgAN/IgAV and the potential necessity for tailored, sometimes combined, treatment strategies. Our case showed an initial unexpected rise in proteinuria before a substantial improvement, ultimately showing lasting benefits with Sparsentan combined with an SGLT2 inhibitor after discontinuing budesonide.

In terms of safety, the PROTECT trial indicated that Sparsentan was generally well-tolerated, showing a safety profile similar to that of irbesartan (10, 19). While AEs like peripheral edema and possible hepatotoxicity were closely monitored (9, 10, 19), Sparsentan was notably well-tolerated in our specific cohort of seven patients. We do acknowledge that there were slightly more frequent elevations in liver enzymes with Sparsentan, in the PROTECT trial, though there were no reported cases of severe drug-induced liver injury (10). But in line with these observations and the prescribing information, Sparsentan includes a boxed warning about hepatotoxicity and mandates monitoring through a Risk Evaluation and Mitigation Strategy (REMS) program. In our cohort of patients, there were no cases of severe hyperkalemia or clinically significant drug-induced liver injury that required stopping the medication, even with concurrent use of SGLT2 inhibitors in five patients, corticosteroids/immunosuppressants in four patients, and a history of MRA use in one patient. Blood pressure was effectively managed, achieving target levels or lower, with no noted symptomatic hypotension that limited continuation of therapy. Regular liver function test monitoring was conducted in our patient cohort according to REMS requirements.

Looking forward, the evolving landscape of IgAN management will likely be defined by multi-targeted regimens. The current wave of promising trials investigating complement-targeted therapies (such as Complement Factor B Inhibitor of the alternative pathway) and B-cell pathway modulators (such as A Proliferation-Inducing Ligand (APRIL) and B cell activating factor (BAFF) inhibitors) will soon offer additional options, and future research should explore the optimal sequence and combination of these agents with established therapies like Sparsentan.

Limitations of this report include its small sample size and observational nature. Treatment regimens varied, and concurrent therapies (e.g., immunosuppressant use, MRA use, and SGLT2 inhibitors) likely influenced the observed outcomes. The follow-up duration is relatively short. Extended follow-up is necessary to assess the impact of Sparsentan-induced reduction in proteinuria on renal function decline, especially in complex cases.

## Conclusion

This case series adds meaningful real-world insights to the literature on Sparsentan, which is mainly based on PROTECT trial data. While reaffirming the significant antiproteinuric benefits observed in clinical studies, this series distinctly demonstrates the use and outcomes of complex clinical scenarios encountered by nephrologists. Notably, it includes insights into: (i) patients with IgA Vasculitis nephritis (excluded from the PROTECT trial) or proliferative IgA glomerulonephritis (such as necrotizing forms); (ii) patients with complex comorbidities and varying degrees of histologic chronicity/activity (including crescents and nephrotic-range proteinuria); and (iii) various combination therapy approaches, such as initial treatment with targeted-release budesonide, addition to ongoing mycophenolate therapy, and simultaneous use with SGLT2 inhibitors. The efficacy observed across these diverse cases, along with a favorable tolerability profile in this cohort (following REMS monitoring), supports Sparsentan’s role as a versatile therapeutic choice beyond the strict definitions of clinical trial populations. These real-world cases emphasize its potential to reduce proteinuria, a crucial aim in mitigating IgAN/IgAV progression, and provide practical instances of its inclusion in comprehensive treatment regimens. Additional research, including larger real-world observational studies and potentially targeted trials, is necessary to confirm and cement these findings, especially concerning optimal combination strategies and long-term renal outcomes among various patient subgroups.

## Data Availability

The original contributions presented in the study are included in the article/**Supplementary Material**. Further inquiries can be directed to the corresponding author.
