# Supplementary material for: Case Report: Maximizing the anti-proteinuric response: a multicenter real-world sparsentan case series in IgA disorders
Source: Front Nephrol. 2025 Nov 21;5:1673799. doi: 10.3389/fneph.2025.1673799 (PMC12679704; doi:10.3389/fneph.2025.1673799)
Supplement: Supplementary file 1 [file Table1.docx]

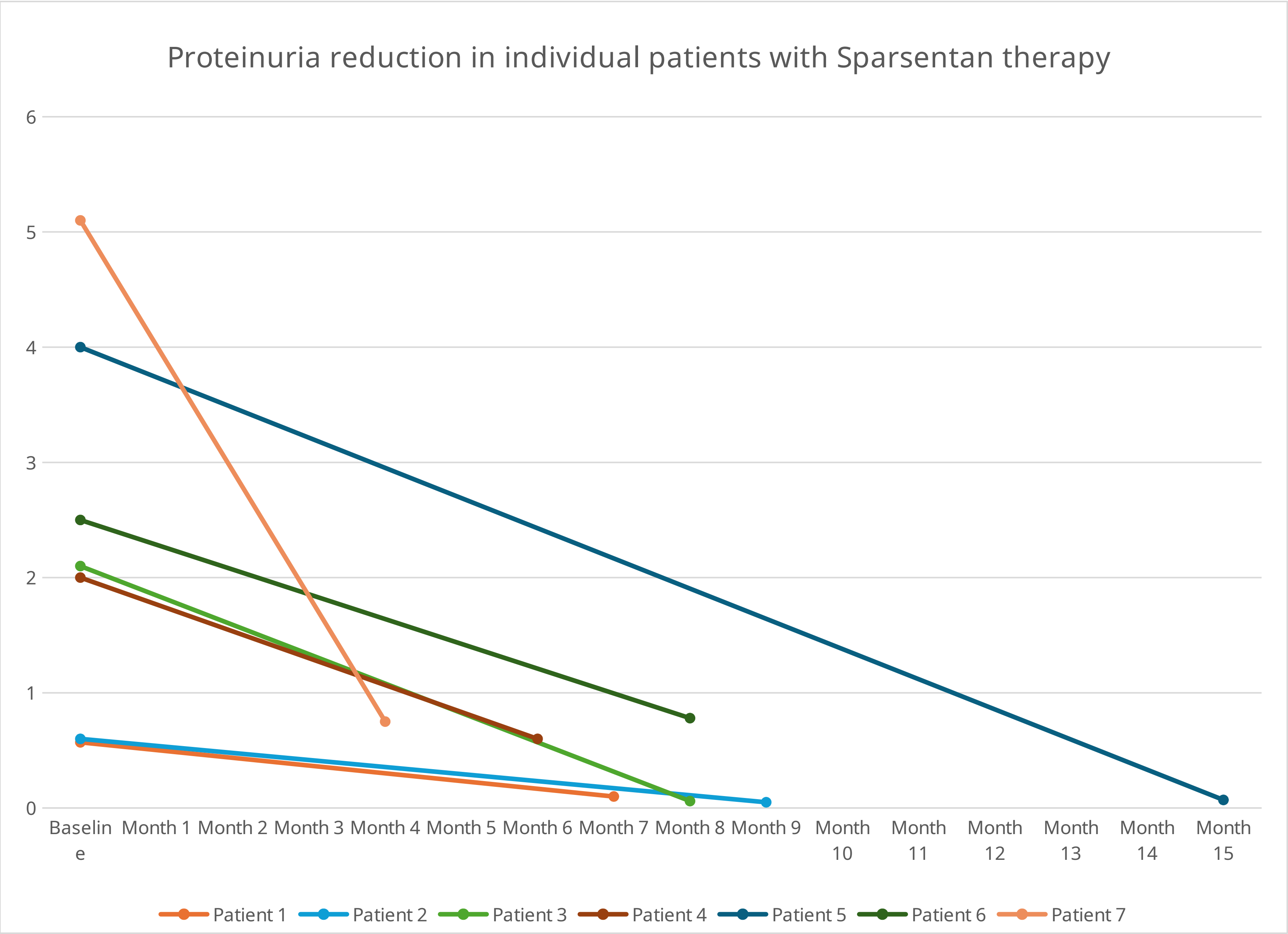

Proteinuria reduction in individual patients with Sparsentan therapy
6
5
4
3
2
1
0
Baseline
Month 1
Month 2
Month 3
Month 4
Month 5
Month 6
Month 7
Month 8
Month 9
Month 10
Month 11
Month 12
Month 13
Month 14
Month 15
Patient 1
Patient 2
Patient 3
Patient 4
Patient 5
Patient 6
Patient 7
